# Bronchoalveolar lavage fluid polymerase chain reaction for invasive pulmonary aspergillosis among high-risk patients: a diagnostic meta-analysis

**DOI:** 10.1186/s12890-023-02343-5

**Published:** 2023-02-07

**Authors:** Yinling Han, Xiang Wu, Guangwei Jiang, Anyi Guo, Zhangchu Jin, Yinghua Ying, Jianxing Lai, Wen Li, Fugui Yan

**Affiliations:** 1 Key Laboratory of Respiratory Disease of Zhejiang Province, Department of Respiratory and Critical Care Medicine, Second Affiliated Hospital of Zhejiang University School of Medicine, Hangzhou, 310009 Zhejiang China; 2 Department of Pulmonary and Critical Care Medicine, Huangshan Hua Ze Hospital of Integrated Traditional Chinese and Western Medicine, Huangshan, 245000 Anhui China; 3 Department of Intensive Care Unit, War Trauma Rescue Center, The 903Rd Hospital of PLA Joint Logistics Support Force, Hangzhou, 310007 Zhejiang China

**Keywords:** Invasive pulmonary aspergillosis, Diagnosis, BAL fluid PCR, Meta-analysis

## Abstract

**Background:**

Polymerase chain reaction (PCR) assays are perceived to facilitate the diagnosis of fungal infections. However, due to lack of standardization, the value of bronchoalveolar lavage (BAL) fluid PCR in diagnosis of invasive pulmonary aspergillosis (IPA) remains unclear.

**Methods:**

We conducted a systematic meta-analysis to evaluate the accuracy of BAL fluid PCR in IPA diagnosis among high-risk patients. All studies involving patients at risk for IPA were included. The sensitivity, specificity, positive and negative likelihood ratios of BAL fluid PCR were summarized for diagnosis of proven/probable IPA, or proven IPA only. Potential heterogeneity was assessed by subgroup analyses and meta-regression.

**Results:**

Forty-one studies involving 5668 patients were analyzed. The summary sensitivity, specificity, positive and negative likelihood ratios of BAL fluid PCR for proven/probable IPA were 0.75 (95% CI = 0.67–0.81), 0.94 (95% CI = 0.90–0.96), 11.8 (95% CI = 7.7–18.1) and 0.27 (95% CI = 0.20–0.36), respectively. Whereas for proven IPA only, sensitivity and specificity were 0.91 (95% CI = 0.68–0.98) and 0.80 (95% CI = 0.74–0.85) in fourteen studies involving 2061 patients. Significant heterogeneity was present due to the underlying disease, antifungal treatment and differences in DNA extraction techniques and choice of PCR assay. Compared to patients with hematological malignancies (HM) and hematopoietic stem cell/solid organ transplantation (HSCT/SOT), sensitivity was higher in the population with disease such as chronic obstructive pulmonary disease, solid tumor, autoimmune disease with prolonged use of corticosteroids, etc. (0.88 vs. 0.68, *P* < 0.001), which was related to the concurrent use of antifungal prophylaxis among patients with HM and HSCT/SOT.

**Conclusion:**

BAL fluid PCR is a useful diagnostic tool for IPA in immunocompromised patients and is also effective for diagnosing IPA in patients without HM and HSCT/SOT. Furthermore, standard protocols for DNA extraction and PCR assays should be focused on to improve the diagnostic accuracy.

*Trial registration* PROSPERO, registration number CRD42021239028.

**Supplementary Information:**

The online version contains supplementary material available at 10.1186/s12890-023-02343-5.

## Introduction

Invasive pulmonary aspergillosis (IPA) is a common opportunistic fungal infection, contributing to high mortality in immunocompromised patients. Early diagnosis of IPA in patients at high risk is essential. It is estimated that more than 200,000 cases of IPA occur every year. The mortality rate of IPA reaches over 50% even if patients are treated with antifungal therapy. Once the diagnosis is delayed or missed, the mortality can be nearly 100% [1]. Traditional diagnostic methods, including histology, cytology, and culture, are time-consuming and have low sensitivity [2, 3].

The molecular diagnostic tool polymerase chain reaction (PCR) is one of the most valuable methods used in diagnosis of respiratory pathogens such as virus and *mycoplasma* [4, 5]. It is also considered as a rapidly expanding technology for fast detection and accurate identification of fungi [6]. Several groups have investigated the performance of PCR from blood or serum in the diagnosis of IPA. A recent meta-analysis performed by Cruciani et al. [7] has assessed the quality of serum PCR for diagnosing IPA from 29 primary studies and concluded that serum PCR showed moderate diagnostic accuracy when used as a diagnostic test. Mengoli et al. [8] has summarized that the diagnostic sensitivity and specificity of blood or serum PCR were 0.75 and 0.87. Arvanitis et al. [9] also finds the similar results.

Bronchoalveolar lavage (BAL) fluid is likely to be more sensitive in early diagnosis [10]. PCR from BAL fluid is recommended for screening the diagnosis of IPA in latest clinical practice guideline from official American Thoracic Society (ATS) and revised European Organization for Research and Treatment of Cancer/Mycoses Study Group (EORTC/MSG) definition [11, 12]. However, both guidelines mainly aim at the diagnosis of IPA in patients with hematological malignancies (HM) and hematopoietic stem cell/solid organ transplantation (HSCT/SOT). The value of BAL fluid PCR in IPA diagnosis among patients with other diseases, such as chronic obstructive pulmonary disease (COPD), solid tumor, pulmonary fibrosis, liver cirrhosis, diabetes, autoimmune disease with prolonged use of corticosteroids (at therapeutic doses ≥ 0.3 mg/kg for ≥ 3 weeks within the past 60 days), treatment with T-cell or B-cell immunosuppressants, ICU admission, etc., remains unclear. Due to lack of standardization, PCR assays vary with respect to DNA extraction protocols, gene targets and amplification platforms, leading to the uncertainty of diagnostic accuracy. Furthermore, several randomized controlled trials (RCT) about BAL fluid PCR in IPA diagnosis have been published, and no updated meta-analysis has been done since 2012. Therefore, we performed a systematic meta-analysis of clinical trials to evaluate the accuracy of BAL fluid PCR assay for the diagnosis of IPA among high-risk patients.

## Methods

### Search strategy

Two investigators independently searched for relevant articles published in the PubMed, Web of Science, Embase and Cochrane Central Register of Controlled Trials databases up to June 2022. Search terms contained “aspergil*”, “PCR”, “bronchoalveolar lavage”, “respiratory” and “sputum”. The syntax was as follows: ((bronchoalveolar lavage) OR (sputum) OR (respiratory)) AND (PCR) AND (aspergil*). The references of included review articles or identified trials were also checked. Searches were restricted to English language literature on human subjects. Duplicate articles identified in mentioned databases were manually deleted.

### Inclusion criteria and definitions

Full-text publications using PCR on BAL fluid were included if (1) they used EORTC/MSG criteria [2, 3, 12] or similar criteria if studies were published before the publication in 2002 for the diagnosis of IPA. (2) they provided data about true-positive, false-positive, false-negative and true-negative results, and (3) the studies included immunocompromised or at-risk patients. Based on EORTC/MSG or similar criteria, patients were classified into four groups on IPA diagnosis: proven, probable, possible and no IPA. We defined the true positive cases if they were classified as proven or probable IPA. Possible IPA cases were excluded because they were considered not reliable enough in the clinical management [13].

### Data collection and risk of bias assessment

The first selection was carried out on the basis of the title and abstract by two investigators. The full paper of each potentially eligible study was then obtained. Two investigators independently assessed eligible studies for inclusion. The relevant information was collected from each selected studies including: first author, year of publication, country, sample size, mean age, prevalence of IPA, percentage of patients with HM and HSCT/SOT in the study population, study design, reference standard, DNA extraction, PCR technology, primers and antifungal therapy. Disagreements between authors were resolved by consensus through group discussion. We assessed the quality of studies by the QUADAS-2 (Quality Assessment of studies of Diagnostic Accuracy included in Systematic reviews) checklist [14] to test potential bias in all studies.

### Outcome of interests

The primary outcomes of interest were the summary sensitivity and specificity of *Aspergillus* PCR in BAL fluid for high-risk patients. Secondly, we aimed to evaluate the effect on heterogeneity among different studies about several critical parameters, such as proportion of patients with HM and HSCT/SOT in the study population, the use of antifungal treatment at time of BAL, method of DNA extraction, PCR technology, etc.

### Data analysis

The sensitivity and specificity of all studies were calculated by constructing two-by-two tables (proven or probable IPA versus possible or no IPA, proven IPA versus probable, possible or no IPA). All the tables included true-positive, false-positive, false-negative and true-negative results of *Aspergillus* PCR assay. We calculated the pooled sensitivity, specificity, likelihood ratios and diagnostic odds ratio (DOR) by random-effect model [15]. Summary receiver operating characteristic curves (SROC) was constructed and diagnostic accuracy (area under the curve, AUC) was estimated [16]. Publication bias was assessed by using the Deeks’ regression test for asymmetry [17]. We assessed statistically heterogeneity by the employment of *I*^2^ statistic [18]. Potential heterogeneity was estimated by meta-regression and subgroup analyses [19] for sensitivity and specificity. The Pearson's *r* was applied to measure the linear correlation between the proportion of patients with HM and HSCT/SOT in the study population and the application of antifungal treatment. All analyses were performed using STATA software version 15 (Stata Corp, College Station, Texas) with the program “midas”. *P* values of < 0.05 denoting statistical significance.

## Results

### Eligible study characteristics

**Fig. 1:**
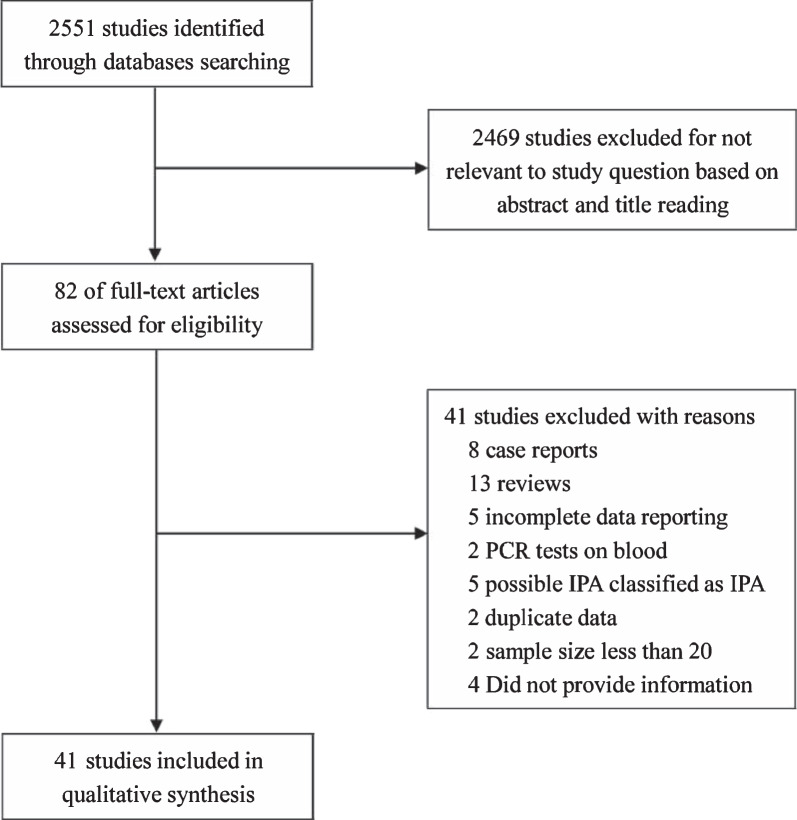
Flow diagram. *PCR* Polymerase chain reaction; *IPA* Invasive pulmonary aspergillosis

The study identified 2551 references by the initial search. Eighty-two were selected based on abstract and title search. Forty-one studies, including 5668 patients, satisfied our inclusion criteria and were in the final analysis [20–60] (Fig. 1).

Table 1 summarized the main characteristics of all included studies. Forty-one studies comprised 17 prospective and 24 retrospective ones, of which 6 were case–control studies. Among all studies, the prevalence of proven/probable IPA ranged from 5 to 51%. Most patients suffered HM. Twelve studies involved 1147 patients mainly with COPD, solid tumor, autoimmune disease with prolonged use of corticosteroids, treatment with T-cell or B-cell immunosuppressants, etc., rather than HM or HSCT/SOT. Fourteen studies containing 2061 patients provided data about proven IPA only. Antifungal intervention against IPA before BAL was definitely described in 20 studies. Details of PCR techniques were summarized in Table 2. Two or three different PCR methods were used in 2 studies respectively. Quality assessment is shown according to the QUADAS-2 (Additional file 1: Fig. S1).

**Table 1:** Main characteristics of studies included

Study	Country	Study population (%)	Mean age	Study design	Criteria	Sample size (n)	Antifungal intervention (n)	Proven/probable IPA (n (%))
Subhagan [20]	India	HM (0)	Unknown	Retrospective cohort	2020^a^	30	0	10 (33)
Mikulska [21]	Italy	HM (< 50)	64 (30–82)	Retrospective cohort	2020	111	Unknown	31 (28)
Scharmann [22]	German	HM (> 50)	61 (18–92)	Retrospective cohort	2020	93	44	10 (11)
Unterman [23]	Israel	HM (0)	53.5 ± 16.3	Retrospective cohort	2008^b^	95	59	5 (5)
Pelzer [24]	German	HM (100)	Unknown	Prospective cohort	2008	100	100	23 (23)
Mikulska [25]	Italy	HM (> 50)	54 (20–81)	Retrospective cohort	2008	123	36	30 (24)
Hardak [26]	Israel	HM (> 50)	55	Retrospective cohort	2008	1248	31	287 (23)
Wehrle-Wieland [27]	Switzerland	HM (100)	57 (21–87)	Prospective cohort	2008	167	53	33 (20)
Prattes [28]	Austria	HM (< 50)	65	Retrospective case–control	Similar^d^	35	Unknown	18 (51)
Heldta [29]	Austria	HM (100)	55	Prospective cohort	2008	101	85	11 (11)
Guegan [30]	France	HM (> 50)	62	Prospective cohort	2008	387	Unknown	38 (10)
Grancini [31]	Italy	HM (< 50)	51.3 (7–83)	Retrospective case–control	2008	110	Unknown	21 (19)
Denis [32]	France	HM (> 50)	Unknown	Retrospective cohort	2008	73	29	31 (42)
Boch [33]	German	HM (< 50)	Unknown	Prospective cohort	2008	44	Unknown	9 (20)
Montesinos [34]	Belgian	HM (< 50)	Unknown	Retrospective cohort	2008	100	Unknown	29 (29)
Eigl [35]	Austria	HM (100)	58	Prospective cohort	2008	72	46	16 (22)
Bhimji [36]	Canada	HM (0)	Unknown	Prospective cohort	Similar	201	Unknown	23 (11)
Zhang [37]	China	HM (0)	Unknown	Retrospective cohort	2008	90	Unknown	10 (19)
Chong [38]	Dutch and Belgian	HM (100)	56.6 (17.5–82.6)	Retrospective cohort	2008	201	Unknown	52 (26)
Boch [39]	German	HM (> 50)	Unknown	Prospective cohort	2008	99	51	43 (43)
Chong [40]	Nehterlands	HM (< 50)	Unknown	Retrospective cohort	2008	77	0	22 (29)
Hoenigl [41]	Austria and German	HM (> 50)	58 (24–77)	Prospective cohort	2008	67	Unknown	10 (15)
Heng [42]	Australia	HM (100)	Unknown	Retrospective cohort	2008	116	79	18 (16)
Reinwald [43]	German	HM (100)	Unknown	Prospective cohort	2008	76	65	29 (38)
Reinwald [44]	German	HM (100)	56	Retrospective cohort	2008	226	146	48 (21)
Buess [45]	Switzerland	HM (> 50)	50.5	Prospective cohort	2008	191	111	11 (6)
Torelli [46]	Italy	HM (< 50)	Unknown	Prospective cohort	2008	158	Unknown	17 (11)
Luong [47]	America	HM (0)	58.4	Retrospective cohort	Similar	150	75	16 (11)
Hadrich [48]	Tunisia	HM (100)	Unknown	Prospective case–control	2008	163	Unknown	44 (27)
Fréalle [49]	France	HM (100)	49	Retrospective cohort	2002^c^	57	> 50%	25 (44)
Shahid [50]	India	HM (0)	Unknown	Prospective case–control	2002	69	0	23 (33)
Khot [51]	America	HM (100)	53.68	Retrospective cohort	2002	81	Unknown	13 (16)
Musher [52]	America	HM (100)	Unknown	Retrospective case–control	2002	93	34	46 (49)
Sanguinetti [53]	Italy	HM (100)	Unknown	Retrospective cohort	2002	44	Unknown	20 (45)
Rantakokko [54]	Finland	HM (100)	Unknown	Retrospective cohort	2002	66	Unknown	11 (174)
Raad [55]	America	HM (< 50)	50	Prospective cohort	Similar	249	Unknown	32 (13)
Hayette [56]	Belgium	HM (< 50)	Unknown	Retrospective cohort	Similar	74	Unknown	10 (14)
Buchheidt [57]	German	HM (> 50)	Unknown	Prospective cohort	Similar	176	Unknown	31 (19)
Jones [58]	British	HM (100)	Unknown	Retrospective cohort	Similar	69	Unknown	12 (17)
Bretagne [59]	France	HM (> 50)	Unknown	Prospective cohort	Similar	52	Unknown	3 (6)
Tang [60]	British	HM (> 50)	Unknown	Retrospective case–control	Similar	51	Unknown	4 (8)

*IPA* Invasive pulmonary aspergillosis; *HM* Hematological malignancy

^a^Studies used revised European Organization for the treatment of Cancer/Mycoses Study Group (EORTC/MSG) criteria [12]

^b^Studies used revised European Organization for the treatment of Cancer/Mycoses Study Group (EORTC/MSG) criteria [3]

^c^Studies used EORTC/MSG criteria [2]

^d^Studies used criteria similar but not identical to the EORTC/MSG criteria

**Table 2:** Technical details of the PCR methods used in the studies included

Study	Sample volume (ml)	Cell wall disruption	DNA isolation	PCR method	Primer
Subhagan [20]	1	Proteinase	Phenol–chloroform	Pan *Aspergillus*	18S rRNA
Mikulska [21]	0.2	QIAamp DSP virus spin kit	QIAamp	Multiplex real-time	28S rRNA
Scharmann [22]	0.5	Maxwell16 tissue LEV total DNA/RNA purification kit	Maxwell16 tissue LEV total DNA/RNA purification	MycoGENIE or Fungiplex or AsperGenius real-time	28S rRNA
Unterman [23]	Unknown	QIAamp DNA mini Kit	QIAamp	Nested pan-*Aspergillus*	18S rRNA
Pelzer [24]	1	Maxwell16 DNA kit	Maxwell16	AsperGenius real-time	*Aspergillus*-species
Mikulska [25]	0.5	MycoGENIE DNA extraction kit	MycoGENIE	MycoGENIE real-time	28S rRNA
Hardak [26]	5	Proteinase	QIAamp	Nested	18S rRNA
Wehrle-Wieland [27]	0.2	Proteinase	EZ1 DNA tissue kit	In-house	ITS1-5.8S rRNA
Prattes [28]	0.4	NucliSens easyMAG	NucliSens easyMAG	MycoGENIE real-time	28S rRNA
Heldta [29]	1	Proteinase	Phenol–chloroform	Nested	*Aspergillus*-species
Guegan [30]	1	Proteinase	QIAamp	In-house	28S rRNA
Grancini [31]	0.2	Proteinase	EZ1 DPS virus kit	Real-time	rDNA18S
Denis [32]	0.2	QIAamp DNA mini kit	QIAamp	Real-time	ITS1 region or 28S rRNA
Boch [33]	1.5	Proteinase	Phenol–chloroform	Nested	18S rRNA
Montesinos [34]	0.8	QIAsymphony DSP virus/pathogen midi kit	QIAsymphony DSP virus/pathogen midi kit	Real-time	*Aspergillus*-species
Eigl [35]	1.5	Proteinase	Phenol–chloroform	Nested	18S rRNA
Bhimji [36]	Unknown	Unknown	Unknown	Droplet digital	Pan-*Aspergillus*
Zhang [37]	1	Glass beads	DNeasy plant mini kit	Real-time	28S rRNA
Chong [38]	1	Proteinase	NucliSENS miniMAG	AsperGenius real-time	28S rRNA
Boch [39]	Unknown	Proteinase	Phenol–chloroform	Nested	18S rRNA
Chong [40]	1	Proteinase	NucliSENS miniMAG	AsperGenius real-time	28S rRNA
Hoenigl [41]	1.5	Proteinase	Phenol–chloroform	Nested	18S rRNA
Heng [42]	0.6	GeneElute mammalian DNA extraction kit	GeneElute mammalian DNA extraction kit	Nested	18S rRNA
Reinwald [43]	1.5	Proteinase	Phenol–chloroform	Nested	18S rRNA
Reinwald [44]	1.5	Proteinase	Phenol–chloroform	Nested	18S rRNA
Buess [45]	2.5	Proteinase	EZ1 DNA tissue Kit	Nested	18S rRNA
Torelli [46]	2	Glass beads	MycXtra fungal DNA	Real-time	18S rRNA
Luong [47]	0.5	Glass beads	AllPrep DNA/RNA mini kit	Real-time	Pan-*Aspergillus*
Hadrich [48]	0.2	Proteinase	QIAamp	Real-time	18S rRNA
Fréalle [49]	0.2	QIAamp DNA mini kit	QIAamp	Real-time	Unknown
Shahid [50]	0.4	Proteinase	Phenol–chloroform	End-point	*Aspergillus*-species
Khot [51]	2–5	MasterPure yeast DNA kit	MasterPure yeast DNA kit	Real-time	18S rRNA
Musher [52]	0.5	Proteinase	MasterPure yeast DNA kit	Real-time	18S rRNA
Sanguinetti [53]	1.5	DNeasy plant mini kit	DNeasy plant mini kit	Real-time	18S rRNA
Rantakokko [54]	1.5	Proteinase	Phenol–chloroform	Real-time	mtDNA
Raad [55]	1	Proteinase	Phenol–chloroform	End-point	alkaline protease mtDNA
Hayette [56]	0.5	Proteinase	Phenol–chloroform	Nested	alkaline protease mtDNA
Buchheidt [57]	1.5	Lyticase	Phenol–chloroform	Nested	18S rRNA
Jones [58]	0.2	Proteinase	Phenol–chloroform	PCR-ELISA	mtDNA
Bretagne [59]	1.5	Proteinase	Phenol–chloroform	Competitive	mtDNA
Tang [60]	0.25	Proteinase	Phenol–chloroform	End-point	alkaline protease mtDNA

*PCR* Polymerase chain reaction; *ITS1* Internal transcribed spacer 1; *rRNA* Ribosomal RNA; *mtDNA* Mitochondrial DNA

### Pooled diagnostic performance for proven/probable IPA in all patients

**Fig. 2:**
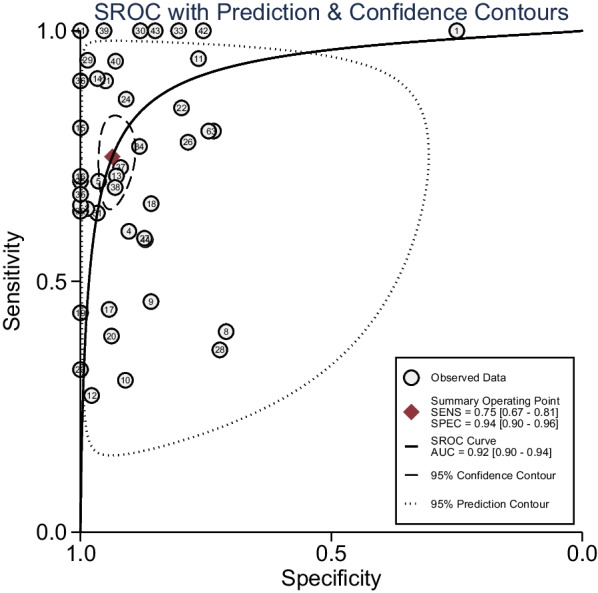
Summary receiver operating characteristic curve of PCR from BAL fluid. The smaller regions (confidence contours) contain possible combinations of sensitivity and specificity means. Broader regions (prediction contours) indicate more uncertainty as to where the likely values of sensitivity and specificity for individual studies might arise

**Fig. 3:**
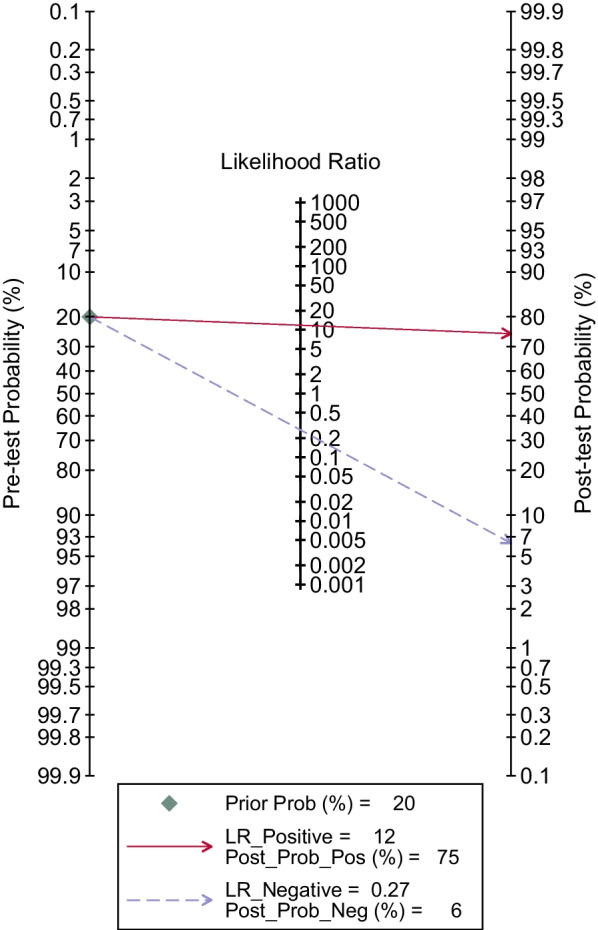
Fagan’s nomogram for calculating post-test probabilities in all studies. Straight edges were used to relate the pretest probability of IPA to post-test probabilities by crossing the likelihood ratio line at the point describing the obtained outcome. Solid lines extend from prevalence to PLR and dashed lines extend from prevalence to NLR

The summary pretest probability of disease was 20%. The sensitivity and specificity with 95% confidence interval for proven/probable IPA (Additional file 1: Fig. S2) were 0.75 (0.67–0.81) and 0.94 (0.90–0.96). Positive and negative likelihood ratios (PLR and NLR) were 11.8 (7.7–18.1) and 0.27 (0.20–0.36). DOR was 44 (25–77). *I*^2^ was more than 50%, which indicated significant heterogeneity was present. The AUC was 0.92 (0.90–0.94) (Fig. 2). The post-test probability indicated that when pretest probability was 20%, PCR method increased the probability to 75% for IPA when the results were positive and decrease to 6% when negative (Fig. 3).

### Pooled diagnostic performance for proven IPA only

Fourteen studies containing 2061 patients described sufficient data for two-by-two table about proven IPA. The pooled sensitivity, specificity (Additional file 1: Fig. S3), PLR, NLR, DOR, AUC with 95% confidence interval were 0.91 (0.68–0.98), 0.80 (0.74–0.85), 4.6 (3.4–6.1), 0.11 (0.03–0.47), 41 (9–193) and 0.89 (0.86–0.91), respectively. The sensitivity for proven IPA was better than that of proven/probable IPAs, whereas the specificity was lower.

### Heterogeneity and publication bias

**Fig. 4:**
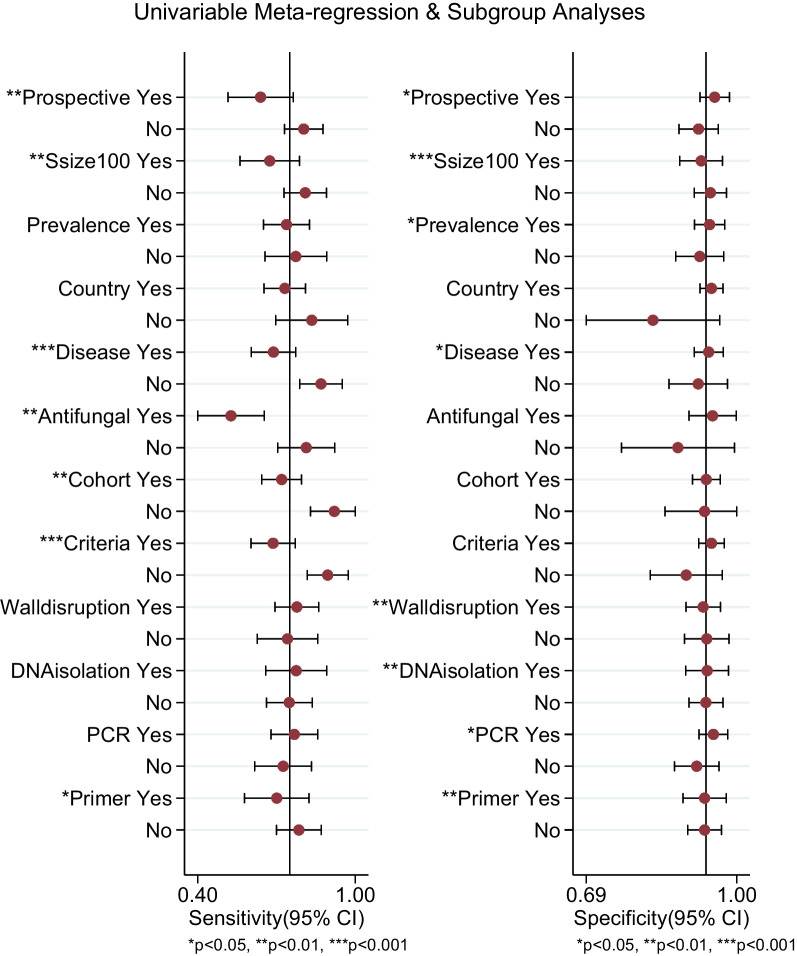
Forest plot of meta-regression and subgroup analyses for sensitivity and specificity. Ssize100: sample size > 100; Prevalence: prevalence above 15%; Country: European countries; Disease: percentage of patients with hematological malignancy above 50%; Criteria: revised EORTC/MSG criteria in 2008; Walldisruption: proteinase for cell wall disruption; DNAisolation: phenol–chloroform for DNA isolation protocol; PCR: real-time PCR; Primer: 18S rRNA primer

Univariable meta-regression and subgroup analyses were estimated for investigating the heterogeneity in all studies (Fig. 4). Subgroup analyses showed that the underlying diseases and the use of antifungal treatment had a significant impact on the diagnostic sensitivity of BAL fluid PCR. Twenty-nine studies involving patients mostly with HM and HSCT/SOT were enrolled. The summary estimates of BAL fluid for proven/probable IPA were as follows (Table 3): sensitivity 0.68 (0.58–0.76), specificity 0.94 (0.89–0.97), PLR 11.3 (6.3–20.3), NLR 0.34 (0.25–0.45), DOR 33 (16–69), AUC 0.89 (0.86–0.92). For patients with disease such as COPD, solid tumor, autoimmune disease with prolonged use of corticosteroids, treatment with T-cell or B-cell immunosuppressants, etc., the pooled sensitivity, specificity, PLR, NLR, DOR, AUC were 0.88 (0.75–0.95), 0.92 (0.83–0.96), 11.0 (5.4–22.7), 0.13 (0.05–0.29), 88 (33–237), 0.96 (0.94–0.97). The diagnostic sensitivity of BAL fluid PCR was much higher in patients without HM and HSCT/SOT (*P* < 0.001). Moreover, use of antifungal agents notably lower the sensitivity of PCR. The antifungal treatment had a strong correlation with the underlying diseases. Pearson correlation coefficient between the proportion of patients with HM and HSCT/SOT in the study population and the application of antifungal treatment was 0.76 (*P* < 0.001).

**Table 3:** Results of subgroup analyses

	DOR (95% CI)	AUC (95% CI)	Sensitivity (95% CI)	Specificity (95% CI)	Likelihood ratio (95% CI)	
					Positive	Negative
Patients with HM and HSCT/SOT	33 (16–69)	0.89 (0.86–0.92)	0.68 (0.58–0.76)	0.94 (0.89–0.97)	11.3 (6.3–20.3)	0.34 (0.25–0.45)
Patients with COPD, solid tumor, prolonged use of corticosteroids, etc	88 (33–237)	0.96 (0.94–0.97)	0.88 (0.75–0.95)	0.92 (0.83–0.96)	11.0 (5.4–22.7)	0.13 (0.05–0.29)

*HM* Hematological malignancy, *HSCT/SOT* Hematopoietic stem cell/solid organ transplantation, *COPD* Chronic obstructive pulmonary disease

Besides, we found some covariates such as study types, group size, prevalence, criteria, DNA extraction protocols, PCR method and primers affected the sensitivity and/or specificity. It was shown sensitivity was lower in prospective, cohort, small group studies and those using revised EORTC/MSG criteria [3]. When DNA isolation kit was used for cell wall disruption, the specificity was a little higher and real-time PCR presented higher specificity whereas the primer 18S rRNA presented lower sensitivity than others. There was significant publication bias in all studies (Additional file 1: Fig. S4).

## Discussion

The article evaluated the value of BAL fluid PCR in diagnosis of IPA. To the best of our knowledge, this is the first meta-analysis focusing on the diagnostic value of BAL fluid PCR for IPA patients with disease such as COPD, solid tumor, autoimmune disease with prolonged use of corticosteroids, treatment with T-cell or B-cell immunosuppressants, etc., rather than HM and HSCT/SOT. In our meta-analysis, the overall sensitivity and specificity was 0.75 and 0.94, respectively in all 41 studies. For proven IPA only, sensitivity was higher, which increased to 0.91, but specificity decreased to 0.80. Twelve studies included patients without HM and HSCT/SOT. The pooled sensitivity of BAL fluid PCR for proven/probable IPA for these 12 studies was 0.88, significantly higher than that among patients with HM or HSCT/SOT (0.68), which was related to the high frequency use of antifungal agents. Different factors containing study types, group size, prevalence, percentage of patients with HM and HSCT/SOT in the study population, use of antifungal agents, DNA extraction, PCR methods and primers were responsible for heterogeneity of included studies. Quality items had no significant influence on diagnostic characteristics. However, there was significant publication bias in our study.

Overall, our analysis showed that BAL fluid PCR was an effective test for IPA diagnosis, especially for patients without HM and HSCT/SOT. In patients with HM and HSCT/SOT, the results of specificity, DOR and AUC of PCR for proven/probable IPA diagnosis were reliable, indicating the test had a good discriminative ability. However, the sensitivity and NLR results were not satisfactory, which could increase the rate of missed diagnosis. But when patients with HM and HSCT/SOT were mainly excluded, sensitivity became higher.

To better explore the diagnostic capacity of PCR in BAL fluid, we made analysis for proven IPA separately. In contrast to the diagnosis of probable IPA depending on host, clinical and mycological factors, the criteria for proven IPA involves histopathologic, cytopathologic, direct microscopic examination or culture from sterile material [2, 3, 12], which could be considered as “gold standard” for IPA diagnosis. Consequently, the analysis for proven IPA only seems to be more objective and we gained higher sensitivity for BAL fluid PCR from proven IPA only, increasing the reliability of the diagnostic test. In addition, the decreased specificity of PCR for proven IPA may be due to the incorrect exclusion of those probable cases who were actually infected.

Significant heterogeneity was present in our analysis. Therefore, we implemented subgroup analyses and meta-regression to search for reasons behind these inconsistencies. It was found that studies comprising patients without HM and HSCT/SOT had higher sensitivity. Patients with HM and HSCT/SOT were tend to be treated with azoles prophylaxis more frequently, especially among HSCT/SOT and neutropenic patients [61], which could explain why the sensitivity was lower in studies whose percentage of patients with HM and HSCT/SOT was high. In our analysis, Pearson correlation coefficient was applied to evaluation the relationship between the proportion of patients with HM and HSCT/SOT and the application of antifungal treatment. We found a strong correlation between antifungal therapy and underlying disease. Use of empiric antifungal agents could influence the summary evaluation, which was confirmed to impact the release of *Aspergillus* DNA, thus lower the residual fungal burden in lung tissue and therefore diminished the sensitivity of PCR assay [62].

Fungal DNA extraction methodology was considered to be the major cause for heterogeneity [63]. Different kinds of DNA extraction methods have been applied in the studies included. The efficiency and the overall performance of wall disruption played a significant role in our analysis as the cell walls of fungi could impede the efficient lysis and liberation of DNA, generating false-negative PCR results [64]. It seemed that commercial nucleic acid extraction methods were more efficient. Besides, the use of DNA isolation mattered as well. Based on these causes, the optimal DNA extraction protocol required verification.

*Aspergillus* species PCR assay may be another reason for variable test performance. Many various PCR amplification protocols have been published, all of whom remain heterogeneous for they lack of standardization. The different methods led to diverse sensitivity or specificity, and we found studies using real-time PCR had better sensitivity and specificity in our analysis. However, according to the European *Aspergillus* PCR initiative (EAPCRI), PCR amplification was not limited and most amplification methods would provide acceptable analytical performance in combination with commercial extraction [65]. What’s more, *Aspergillus* gene targets varied in 18S ribosomal RNA (rRNA), 28S rRNA, the intervening internal transcribed spacer (ITS)-5.8S region, mitochondrial DNA or species-specific primers. A single species, several related species or pan-fungal amplification indicated diverse sensitivity and specificity [63]. In general, because there was so much uncertainty for the use of PCR, the application calls for more standardization.

Compared with BAL galactomannan (GM), PCR from BAL fluid seemed to have superiority in the diagnosis of IPA. Though GM test in plasma, serum or BAL fluid has been incorporated into the EORTC/MSG criteria as one of the clinical diagnostic basis, GM still has its limitations. Affolter et al. reported a moderate diagnostic value of GM in BAL fluid with 50% sensitivity and 73% specificity for proven/probable IPA [66]. For proven IPA only, the specificity decreased, whereas the sensitivity was similar. A meta-analysis conducted by Heng et al. [67] found excellent sensitivity and specificity of GM in BAL fluid for proven/probable IPA. However, when it was estimated for proven IPA only, the specificity decreased to 72%. Similar findings have been reported by Guo et al. [68]. The declining specificity may due to a consequence of false-positive of GM test, increasing the classification of probable IPA using the EORTC/MSG definitions. Besides, the diagnostic accuracy of GM from BAL fluid for patients without HM and HSCT/SOT has not been defined.

There were several limitations in this study. First, the total number of patients without HM and HSCT/SOT included for analysis was relatively small. Second, there was significant heterogeneity in summary estimates. The heterogeneity could be partially explained by study types, group size, the use of antifungal agents, percentage of patients with HM and HSCT/SOT in the study population, DNA extraction, PCR methods and primers. Third, there was significant publication bias in the study. Lastly, misclassification bias could occur when the clinical criteria were used because the EORTC/MSG definition is not the “gold standard” for probable IPA. To identify the accuracy of BAL fluid PCR in IPA diagnosis and its superiority over other methods such as BAL or serum GM test, multicenter RCT study designed with standard criteria is needed.

## Conclusion

In conclusion, PCR from BAL fluid is an effective test in IPA diagnosis, indicating the infection of *Aspergillus* when the result is positive. And the technique seems to be more valuable in the diagnosis of high-risk patients without HM and HSCT/SOT. To improve the accuracy of the test, standardization of DNA extraction and PCR methods is needed for clinical diagnosis.

## Supplementary Information

**Additional file 1: Fig. S1.** Overall quality assessment of included studies (QUADAS-2 tool). **Fig. S2.** Forest plot of sensitivities and specificities of PCR in BAL fluid for proven/probable IPA in all studies. **Fig. S3.** Forest plot of sensitivities and specificities of PCR in BAL fluid for proven IPA only. **Fig. S4.** Deeks’ funnel plot for estimating publication bias.

## Data Availability

Not applicable.

## Abbreviations

AUC: Area under the curve
BAL: Bronchoalveolar fluid
DOR: Diagnostic odds ratio
EORTC: European Organization for Research and Treatment of Cancer
GM: Galactomannan
HM: Hematological malignancy
HSCT/SOT: Hematopoietic stem cell/solid organ transplantation
IPA: Invasive pulmonary aspergillosis
MSG: Mycoses study group
NLR: Negative likelihood ratio
PCR: Polymerase chain reaction
PLR: Positive likelihood ratio
rRNA: Ribosomal RNA
SORC: Summary receiver operating characteristic
