# Supplementary material for: Bronchoalveolar lavage fluid polymerase chain reaction for invasive pulmonary aspergillosis among high-risk patients: a diagnostic meta-analysis
Source: BMC Pulm Med. 2023 Feb 7;23:58. doi: 10.1186/s12890-023-02343-5 (PMC9906844; doi:10.1186/s12890-023-02343-5)
Supplement: Supplementary file 1 — Additional file 1: Fig. S1. Overall quality assessment of included studies (QUADAS-2 tool). Fig. S2. Forest plot of sensitivities and specificities of PCR in BAL fluid for proven/probable IPA in all studies. Fig. S3. Forest plot of sensitivities and specificities of PCR in BAL fluid for proven IPA only. Fig. S4. Deeks’ funnel plot for estimating publication bias. [file 12890_2023_2343_MOESM1_ESM.docx]

**Supplemental Materials**

**Figure S1**

Figure S1 Overall quality assessment of included studies (QUADAS-2 tool)

Figure S2


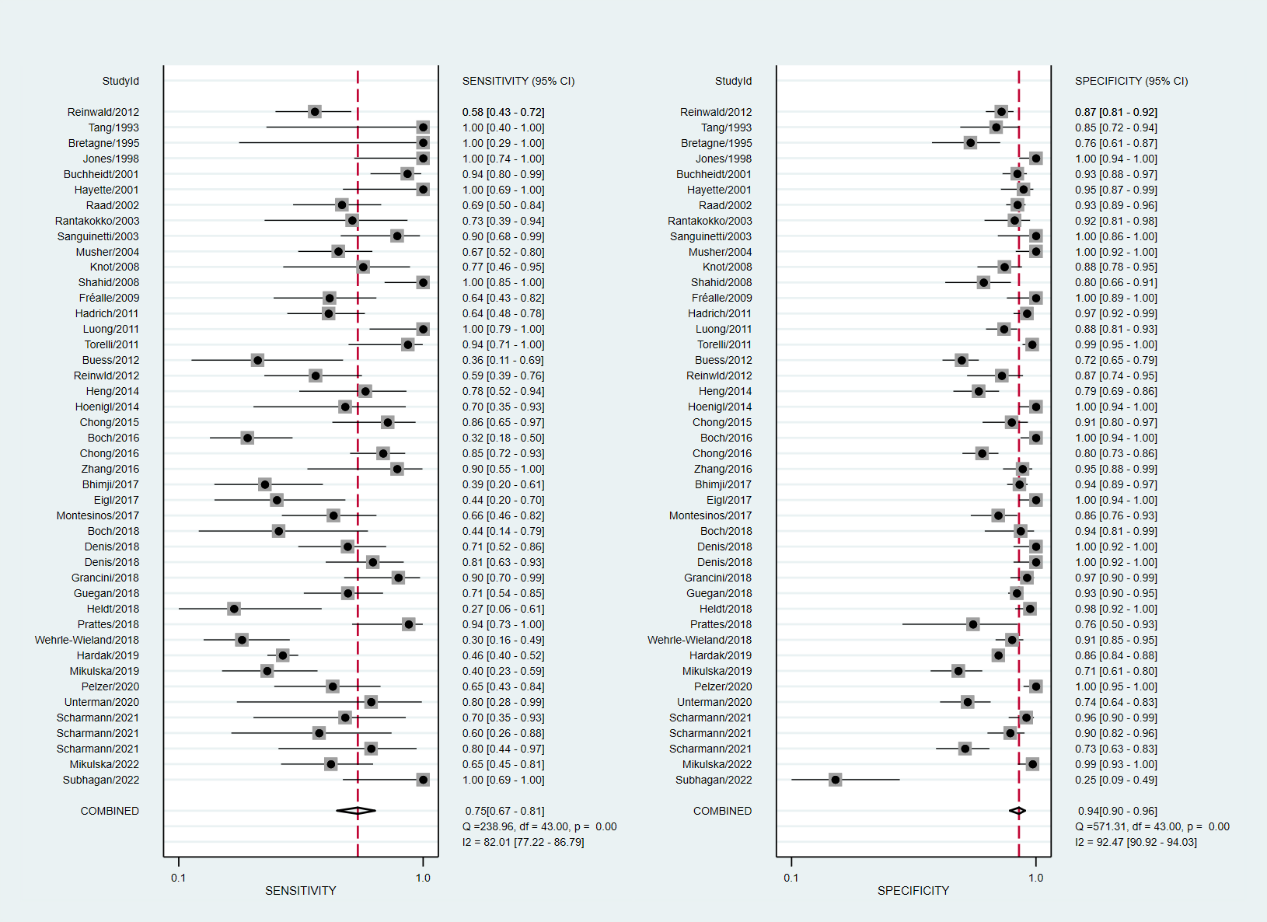


Figure S2 Forest plot of sensitivities and specificities of PCR in BAL fluid for proven/probable IPA in all studies.

Figure S3


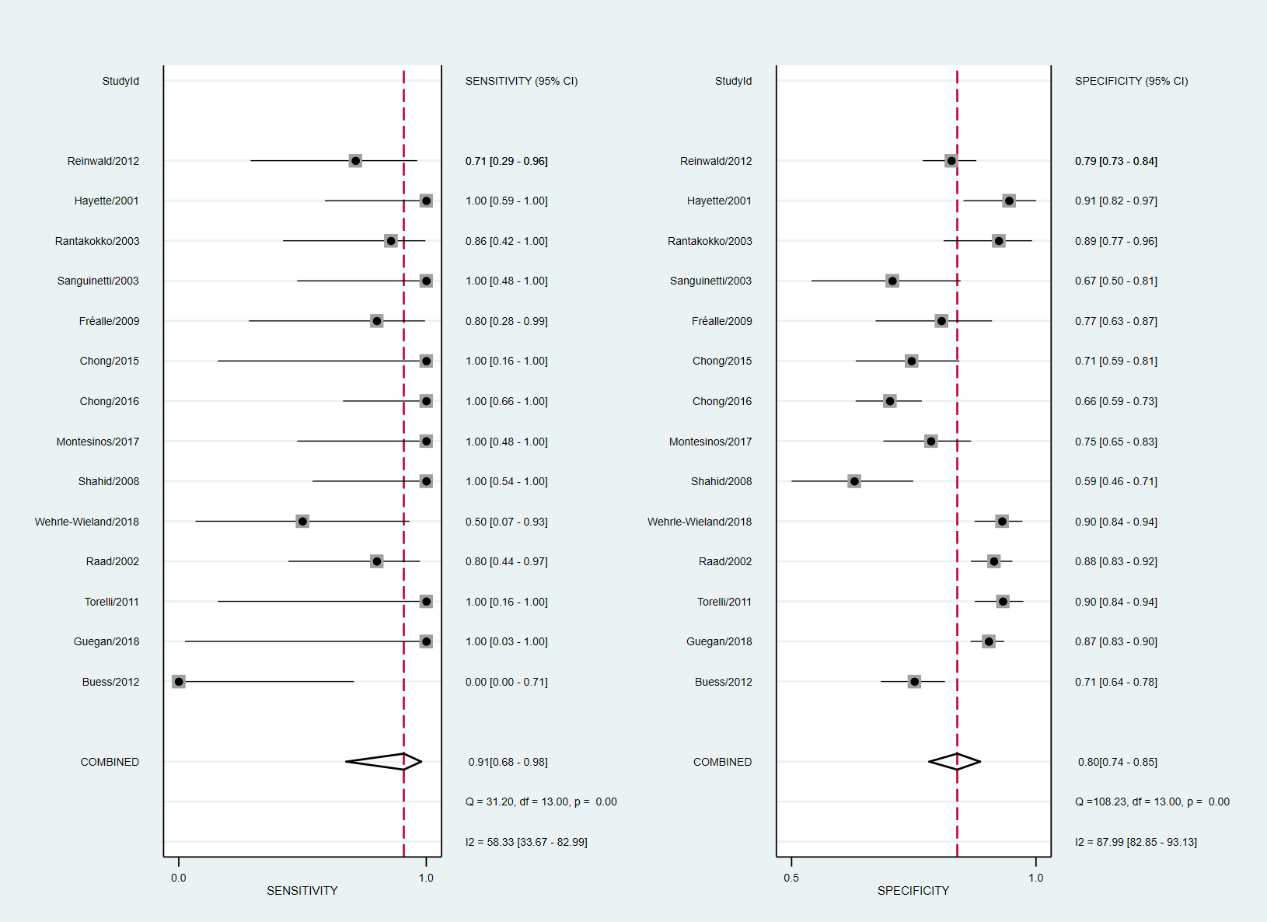


Figure S3 Forest plot of sensitivities and specificities of PCR in BAL fluid for proven IPA only.

Figure S4


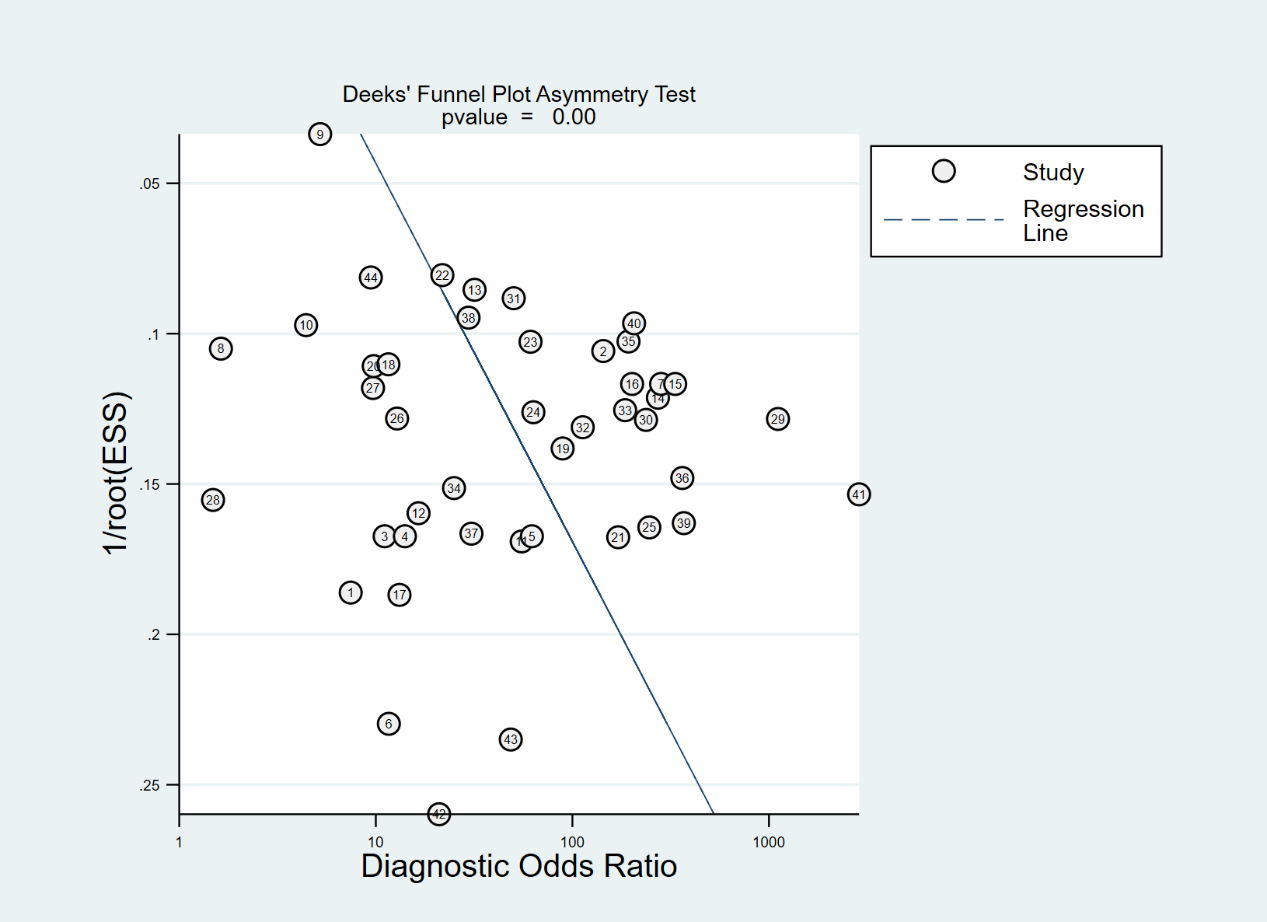


Figure S4 Deeks’ funnel plot for estimating publication bias.
